# YB-1 regulates Sox2 to coordinately sustain stemness and tumorigenic properties in a phenotypically distinct subset of breast cancer cells

**DOI:** 10.1186/1471-2407-14-328

**Published:** 2014-05-09

**Authors:** Karen Jung, Fang Wu, Peng Wang, Xiaoxia Ye, Bassam S Abdulkarim, Raymond Lai

**Affiliations:** 1 Departments of Oncology, Edmonton, AB, Canada; 2 Laboratory Medicine and Pathology, University of Alberta, Edmonton, AB, Canada; 3 Department of Oncology, McGill University, Montreal, QC, Canada; 4 DynaLIFE Dx Medical Laboratories, Edmonton, AB, Canada

**Keywords:** YB-1, Sox2, Breast cancer, Gene transcription, Tumor heterogeneity, Nanog, Cyclin D1, CD49f

## Abstract

**Background:**

Sox2, a transcription factor and an embryonic stem cell marker, has been implicated in the pathogenesis of breast cancer (BC). YB-1 is another transcription factor that has been shown to promote stemness in BC cells.

**Methods:**

Western blotting, quantitative PCR, and siRNAs were used to query the regulatory relationships between YB-1, Sox2, and their downstream targets. Chromatin immunoprecipitation was used to detect YB-1 interactions at the Sox2 promoter. Mammosphere and soft agar assays were used to assess the phenotypic consequences of YB-1 knockdown.

**Results:**

Here, we report that YB-1 regulates Sox2. YB-1 was found to bind to the *SOX2* promoter and down-regulate its expression in MCF7 and ZR751. The regulatory interaction between YB-1 and Sox2 was drastically different between the two phenotypically distinct cell subsets, purified based on their differential response to a Sox2 reporter. They are referred to as the reporter unresponsive (RU) cells and the reporter responsive (RR) cells. Upon siRNA knockdown of YB-1, RU cells showed an increase in Sox2 expression but no change in Sox2 reporter activity; in contrast, RR cells exhibited increased expression and reporter activity of Sox2. Correlating with these findings, YB-1 knockdown induced a differential response in the expression of genes known to be regulated by both Sox2 and YB-1 (e.g. *CCND1* and *ITGA6*). For instance, in response to YB-1 knockdown, *CCND1* and *ITGA6* expression were decreased or unchanged in RU cells but paradoxically increased in RR cells. Compared to RU cells, RR cells were significantly more resistant to the suppression of mammosphere formation due to YB-1 knockdown. Importantly, mammospheres derived from parental MCF7 cells treated with YB-1 siRNA knockdown exhibited higher expression levels of *SOX2* and its downstream targets.

**Conclusions:**

To conclude, in a subset of BC cells, namely RR cells, YB-1 regulates Sox2 to coordinately maintain stemness and tumorigenic properties.

## Background

Sex-determining region Y-box 2 (Sox2) is a transcription factor that plays an important role in maintaining pluripotency in embryonic stem cells [1] and in the generation of inducible pluripotent stem cells [2]. In embryonic stem cells, Sox2 binds to the promoters of many genes, thereby transactivating or suppressing their expressions [3]. In normal adult tissues, the expression of Sox2 is largely restricted to somatic stem cells [4]. In recent years, Sox2 has been found to be aberrantly expressed in cancers, including those of the lungs, brain, ovaries, bone, colon, skin and breasts [5-12]. In many of these tumor types, Sox2 was found in the cancer stem-like cell population [8,13-16]. Studies using various experimental models have demonstrated that Sox2 promotes key tumorigenic properties in cancer cells, including proliferation, invasion, migration, colony formation, non-adherent stem cell-associated sphere formations *in vitro* and tumorigenicity in vivo [8,12-17]. Further, Sox2 expression has been found to correlate with a worse clinical outcome in cancer patients [11,18-20]. In breast cancer (BC), aberrant expression of Sox2 has been found in up to 30% of tumors [11,15], and *in vitro* studies have provided evidence that Sox2 contributes to cell proliferation and mammosphere formation in BC cell lines [12,15].

Similar to Sox2, Y-box binding protein-1 (YB-1) is a transcription factor that has been found in embryonic stem cells, mammary progenitor cells and BC cells [21-23]. Found in 40% of BC tumors [24], YB-1 is believed to promote the tumorigenesis of BC, since it has been shown to enhance mammosphere formation *in vitro*, and transcriptionally up-regulate the expressions of a large cassette of stem cell-associated proteins including CD44, CD49f (α6 integrin), c-Met, EGFR, Her-2, Cyclin D1, MDR-1, and p110α [22,25-28]. ln other cell types, it has been shown that YB-1 can also suppress gene expression, such as those encoding Fas and granulocyte macrophage colony-stimulating factor [29,30]. To mediate gene regulation, YB-1 translocates into the nucleus and interacts with the proximal promoter regions of its target genes [31,32]. YB-1 is activated by phosphorylation in its DNA binding domain, and this biochemical modification confers YB-1 the properties of nuclear translocation and DNA interactions; Akt, RSK1/2 and GSK3ß kinases have been shown to phosphorylate/activate YB-1 [31-33].

Since both YB-1 and Sox2 are important embryonic stem cell proteins that appear to exert similar biological effects in BC [1,23], we hypothesized that they may have important interactions in BC cells. In this regard, we recently found that total YB-1 and phospho-YB-1^Ser102^ (a commonly used surrogate marker of YB-1 activation) are expressed in MCF7 and ZR751, two Sox2-expressing BC cell lines. In this study, we found evidence that YB-1 regulates the expression of Sox2. Importantly, YB-1 exerted different biological effects in the distinct phenotypically distinct cell subsets, separated based on their differential responsiveness to a Sox2 reporter, namely reporter responsive (RR) cells and reporter unresponsive (RU) cells [16]. The biological implications of our findings will be discussed, particularly in the context that RR cells have been previously shown to be more tumorigenic and stem-like as compared to RU cells [16].

## Methods

### Cell lines, cell culture, and reagents

Parental breast cancer cell lines ER + MCF7, ZR751, and ER- MDA-MB-231 were purchased from American Type Culture Collection (ATCC, Rockville, MD). The Unsorted, RU, and RR cell lines are derived as previously described [16]. The Unsorted cells refer to parental cells stably infected with the Sox2 reporter while RU and RR cells have been purified based on GFP expression [16]. Since the Sox2 reporter carries dual genes encoding both luciferase and green fluorescence protein (GFP), RR cells but not RU cells stably show luciferase activity and GFP expression over time [16]. RU and RR cells are maintained and culture separately for our studies, and keep their distinct phenotypes [16]. All the above mentioned cell lines were maintained in high glucose Dulbecco’s Modified Eagle Medium (DMEM) (Life Technologies, Grand Island, NY) supplemented with 10% fetal bovine serum (FBS) (Life Technologies). LY294002 (#L9908, Sigma-Aldrich Canada, Oakville, ON, Canada), SL0101 (#559285, Calbiochem, EMD Millipore Corporation, Billerica, MA), and CHIR99021 (#361559, Calbiochem) were solubilized in DMSO. Insulin-like growth factor-1 (IGF-1) (#I3769, Sigma-Aldrich) was solubilized in phosphate buffered saline (PBS).

### Western blotting

Western blot analyses were performed as previously described [16]. All antibodies were diluted in 5% bovine serum albumin (BSA) in Tris buffered saline and 0.1% Tween-20 (TBST): anti-Sox2 (1:500, #2683-1), and anti-total YB-1 (1:100,000, #2397-1) were purchased from Epitomics, Burlingame, CA. Anti-phospho-YB-1^Ser102^ (1:500, Cat. #2900), anti-phospho-Akt (1:1000, #9271), anti-total Akt (1:1000, #9272), and anti-vinculin (1:1000, #4650) were purchased from Cell Signaling Technologies, Danvers, MA. The expression of vinculin served as the loading control for all western blots.

### siRNA knockdown of YB-1

Two siRNA species, #1 and #2 (corresponding to SI03019191 and SI04172007 respectively, Qiagen Canada, Toronto, Ontario, Canada), were used to knockdown YB-1. The use of scrambled siRNA (ON-TARGETplus Non-targeting Pool, #477C20, Dharmacon, ThermoScientific, Waltham, MA) served as the negative control. For each reaction, 40 pmol of siRNA (20 nM final concentration) and 5 μL of Lipofectamine RNAiMAX (Life Technologies) were added to 0.5 mL of OptiMEM media (Life Technologies), and 800,000 cells in normal culture medium in 6-well plates were reverse transfected. Cells were incubated with siRNAs for 72 or 96 hours before harvesting. We have employed the use of 2 unique siRNA sequences targeted against YB-1. We have primarily used YB-1 siRNA#1 throughout the study as we have achieved successful and consistent knockdowns with this sequence in our laboratory and previous work done by the first author [25]; as well it is the recommended validated sequence from the manufacturer. We have incorporated the use of YB-1 siRNA#2 in our study to validate the findings of YB-1 siRNA#1. In the mammosphere culture condition, we found that the YB-1 siRNA#2 sequence produced a much more robust sustained knockdown 10 days post-transfection and thus we reported the results using the YB-1 siRNA#2 sequence. YB-1 siRNA denotes YB-1 siRNA #1 throughout the manuscript and figures.

### Chromatin immunoprecipitation (ChIP)

ChIP protocol was performed as previously described [16]. Anti-total YB-1 antibody (same as for western blotting) was used at 5 μg per immunoprecipitation. Normal IgG rabbit antibody was used at 5 μg per immunoprecipitation (#2729, Cell Signaling Technologies). ChIP primers sequences were: Sox2 (a): Forward (F) – GAGAGAAAAAGGAGAACCTTCG, Reverse (R) – ACGGTGCATTGTTTTGTTCC; Sox2 (d): F – CCCAACAAGAGAGTGGAAGG, R – ATTTTAGCCGCTCTCCCATT.

### RNA extraction, cDNA synthesis, quantitative reverse transcriptase PCR (q-RT-PCR)

Total RNA extraction was performed with the Qiagen RNeasy Kit (Qiagen Canada) according to the manufacturer’s protocol. Briefly, 1 μg of RNA was reverse transcribed using Superscript II (Life Technologies) according to the manufacturer’s protocol. 1 μL of the resulting cDNA mixture was added to the Platinum SYBR Green qPCR SuperMix-UDG with Rox (Life Technologies) and amplified with target gene specific primers. Primers sequences, Sox2: F – ACAACTCGGAGATCAGCAA, R – GTTCATGTGCGCGTAACTGT; Nanog: F – CTCCAACATCCTGAACCTCAGC, R – CGTCACACCATTGCTATTCTTCG; CD49f: F – ATGCACGCGGATCGAGTTT, R – TTCCTGCTTCGTATTAACATGCT; Cyclin D1: F – GCTGCGAAGTGGAAACCATC, R – CCTCCTTCTGCACACATTTGAA; EGFR: F – GTGACCGTTTGGGAGTTGATGA, R – GGCTGAGGGAGGCGTTCTC; Her2: F – GGGAAGAATGGGGTCGTCAAA, R – CTCCTCCCTGGGGTGTCAAGT. All genes of interest are normalized to GAPDH transcript expression levels, sequence as previously described [16].

### Luciferase assay

The Luciferase Assay System (#E4530, Promega, Corporation, Madison, WI) was performed according to the manufacturer’s protocol, plated on Costar white polystyrene opaque 96-well plates (#3912, Corning, Corning, NY) and analyzed on the FLUOstar Omega multi-mode microplate reader (BMG Labtech, Ortenburg, Germany).

### Flow cytometry

Flow cytometry analyses were performed as previously described [16].

### Mammosphere assay

Mammospheres were seeded and cultured as previously described [16]. Briefly, cells were trypsinized and passed through a 40 μm cell strainer (BD, Franklin Lakes, New Jersey) and seeded into ultra-low adherent plates (Corning) in Mammocult media (StemCell Technologies, Vancouver, BC, Canada). For subsequent RNA extractions, mammospheres were isolated and collected by centrifugation as per manufacturer’s protocol.

### Soft agar colony formation assay

20% 2X DMEM was mixed at a 1:1 ratio with a 1.4% agarose solution (0.7% final concentration) and seeded at 400 μL/well into a 24-well plate. 10,000 cells were mixed with 200 μL 20% 2X DMEM and mixed with 200 μL 0.7% agarose solution (0.35% final concentration) and seeded atop the bottom agarose layer. 10% DMEM media was added on top and changed weekly. Colonies were counted at 14 days.

### Statistical Analyses

Paired Student’s T-tests were used for statistical analyses of experiments throughout, where p < 0.05 is denoted by *, and p < 0.01 is denoted by **. All graphs represent the average of at least 2 independent experiments with triplicates. The error bars represent the standard error of the mean.

## Results

### YB-1 negatively regulates Sox2 expression in breast cancer

**Figure 1:**
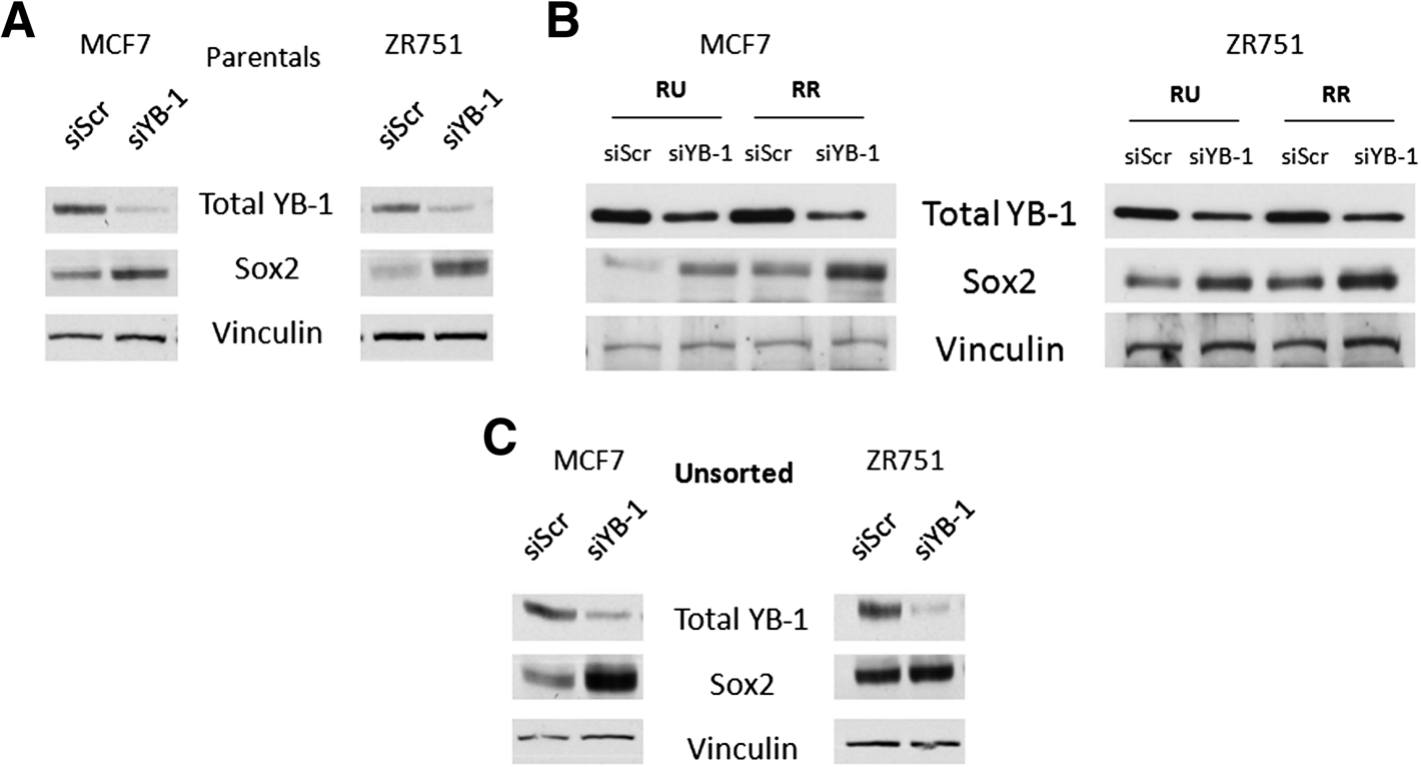
**YB-1 negatively regulates Sox2 protein expression in breast cancer cells. (A)** Western blot of total YB-1 and Sox2 protein expression in MCF7 and ZR751 parental and **(B)** MCF7 and ZR751 RU, RR, and **(C)** Unsorted cells after 72-hour treatment of scrambled or YB-1 siRNA at 20 nM, denoted by siScr and siYB-1.

We first examined if Sox2 expression can be regulated by YB-1. As shown in Figure 1A, we found that siRNA knockdown of YB-1 induced a substantial increase in Sox2 protein expression in parental MCF7 and ZR751, two Sox2-expressing and estrogen receptor (ER)-positive BC cell lines. As we recently discovered that MCF7 and ZR751 are composed of two phenotypically distinct cell subsets that can be separated based on their differential response to a Sox2 activity reporter [16], we asked if YB-1 interacts with Sox2 differently in these two cell subsets, namely RU and RR cells. SiRNA knockdown of YB-1 effectively induced an up-regulation of Sox2 protein expression in both RU and RR cells (Figure 1B). In both the “Unsorted” MCF7 and ZR751 cells, YB-1 knockdown also induced an up-regulation of Sox2 protein expression (Figure 1C). The “Unsorted” cells are the parental cells that have been stably infected with the Sox2 reporter but have not been purified or sorted into RU and RR cells.

It has been previously shown that phospho-YB-1^Ser102^, a surrogate marker of its activation [31,32], is elevated in ER-negative BC [22] than ER-positive BC. Based on our observation that YB-1 down-regulates Sox2, we then predicted that ER-negative BC expresses a lower level of Sox2 than ER-positive BC, due to their higher YB-1 activity. Based on the previously published gene array studies using 50 BC cell lines [34], we found that the expression level of *SOX2* was indeed higher in ER-positive cell lines (Additional file 1: Figure S1). Moreover, in our own study including a small cohort of BC cell lines [16], we did observe a higher Sox2 protein expression in ER-positive cells lines. Taken together, these observations further support that the YB-1 is a negative regulator of Sox2 in BC.

We also asked if Sox2 regulates YB-1. As shown in Additional file 1: Figure S2, siRNA knockdown of Sox2 in MCF7 and ZR751 did not result in any detectable change in the protein expression of total YB-1 or phospho-YB-1^Ser102^.

### YB-1 binds to the SOX2 promoter and regulates Sox2 expression

**Figure 2:**
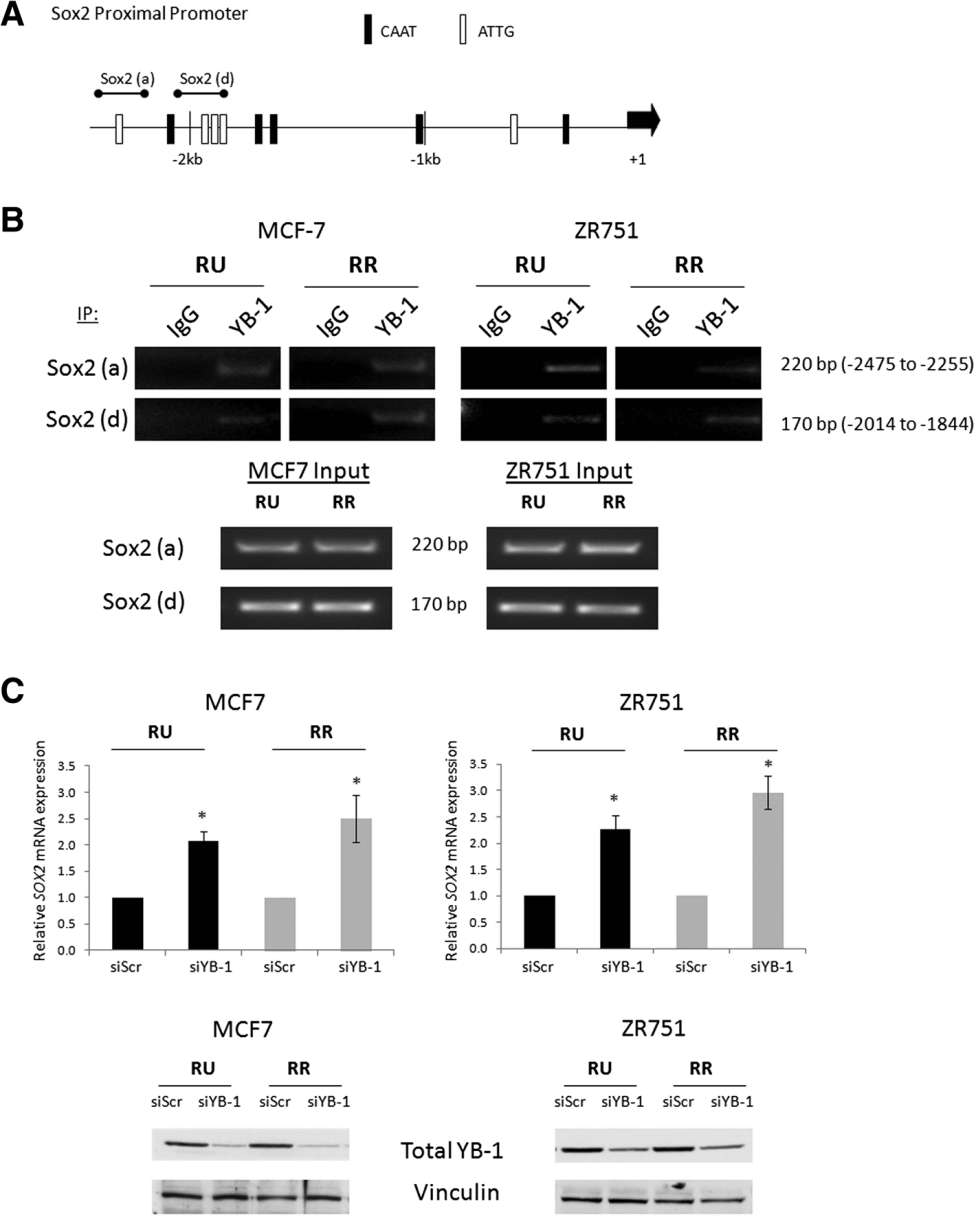
**YB-1 binds to the *SOX2 *promoter and regulates Sox2 transcripts. (A)** Schematic diagram of the Sox2 proximal promoter (2.5 kb upstream of transcriptional start site, denoted by +1) with markings of the minimal YB-1 consensus sequence ATTG/CAAT. Sox2 promoter ChIP primers designed against putative binding sites are shown. **(B)** MCF7 and ZR751 RU and RR ChIP DNA agarose gel results of DNA sequences immunoprecipitated by normal rabbit IgG or a rabbit anti-human YB-1 antibody amplified by Sox2 promoter specific primers a and d. MCF7 Input and ZR751 Input represent the DNA isolated from chromatin before immunoprecipitation to show equal input amounts. **(C)** Quantitative-RT-PCR results illustrating Sox2 mRNA transcript levels after 72-hour 20nM YB-1 siRNA treatment. Western blot shows YB-1 knockdown efficiency.

To examine if YB-1 regulates Sox2 at the transcriptional level, we searched the proximal promoter region of *SOX2* (−1 to −2.5 kb upstream of the transcription start site) for the minimal consensus sequence that confers YB-1 binding, ATTG/CAAT [31]. We identified 10 putative YB-1 binding sites in the *SOX2* promoter (Figure 2A). Using chromatin immunoprecipitation (ChIP) and primers designed to flank these YB-1 putative binding sites, we found evidence that YB-1 binds to the *SOX2* promoter at two adjacent regions (Figure 2B). Further, promoter DNA binding by YB-1 was detectable in both RU and RR cells derived from MCF7 and ZR751 cells (Figure 2B). To further support that YB-1 regulates Sox2 at the transcriptional level, we found that the *SOX2* mRNA transcripts were significantly upregulated in response to siRNA knockdown of YB-1 in both RU and RR cell populations derived from MCF7 and ZR751 (Figure 2C). We also detected this phenomenon in MDA-MB-231, an ER-negative BC cell line (Additional file 1: Figure S3).

### Activation of YB-1 suppresses Sox2 expression

**Figure 3:**
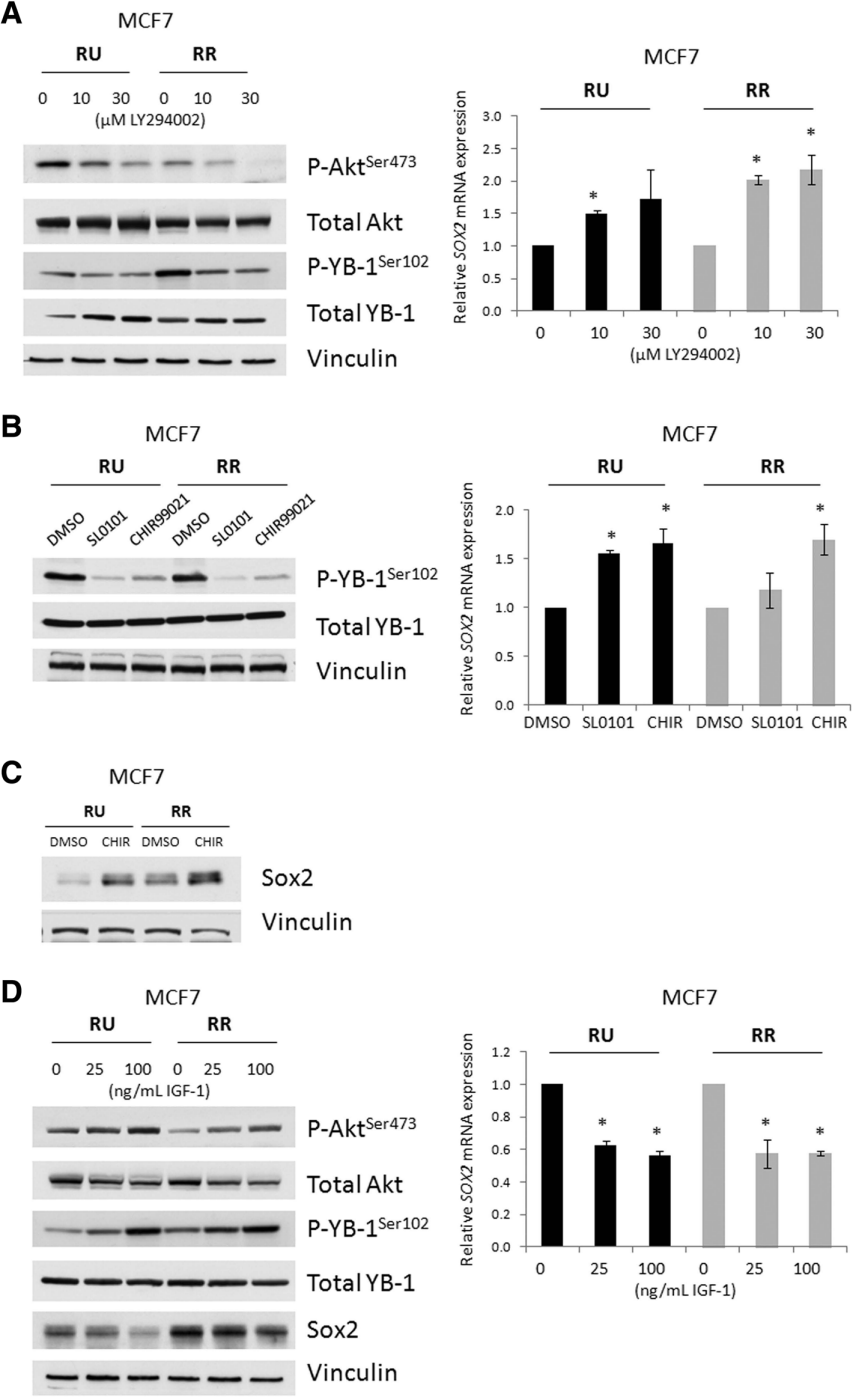
**Activation of YB-1 suppresses Sox2 expression.** Western blots depicting **(A)** altered PI3K/Akt/YB-1 signaling after 0, 10, or 30 μM treatments of LY294002 (PI3K inhibitor) for 6 hours, **(B)** altered YB-1 phosphorylation after 24-hour treatments of 50 μM SL0101 (RSK1/2 inhibitor) and 10 μM CHIR99021 (GSK3ß inhibitor), **(C)** Sox2 protein expression after 72-hour treatment of 10 μM CHIR99021, and **(D)** altered PI3K/Akt/YB-1 signaling after 6-hour 0, 25, or 100 ng/mL IGF-1 stimulations in MCF7 RU and RR cells. Accompanying q-RT-PCR graphs of *SOX2* transcript levels are shown. DMSO only treatments were used as vehicle controls except for **D** where PBS only treatments were used.

To address if YB-1 activation, as evidenced by phosphorylation at its serine-102 residue [31,32], modulates Sox2 expression, we treated MCF7 cells with a small molecule (LY294002) that inhibits activation of Akt, a kinase previously shown to directly phosphorylate YB-1 [31]. As shown in Figure 3A, increasing concentrations of LY294002 down-regulated phospho-YB-1^Ser102^ in a dose-dependent manner and increased the *SOX2* mRNA transcript levels in both RU and RR cells. The same results were obtained with SL0101 and CHIR99021, two small molecule inhibitors of two other direct upstream YB-1 kinases/activators, p90 ribosomal S6 kinase (RSK) 1/2 and glycogen synthase kinase 3-beta (GSK3ß), respectively [32,33] (Figure 3B). Further, we were able to detect an increase in Sox2 protein expression when MCF7 cells were treated for 72 hours with the superiorly stable GSK3ß inhibitor, CHIR99021 (Figure 3C). Conversely, increasing concentrations of insulin-like growth factor-1 (IGF-1), an activating growth factor of the PI3K/Akt/YB-1 pathway, elevated phospho-YB-1^Ser102^ and led to decreased Sox2 protein expression (Figure 3D). These findings support the concept that the activation status of YB-1 is important in regulating Sox2 expression in BC cells.

### YB-1 regulates the Sox2 reporter activity only in the RR cell subset

**Figure 4:**
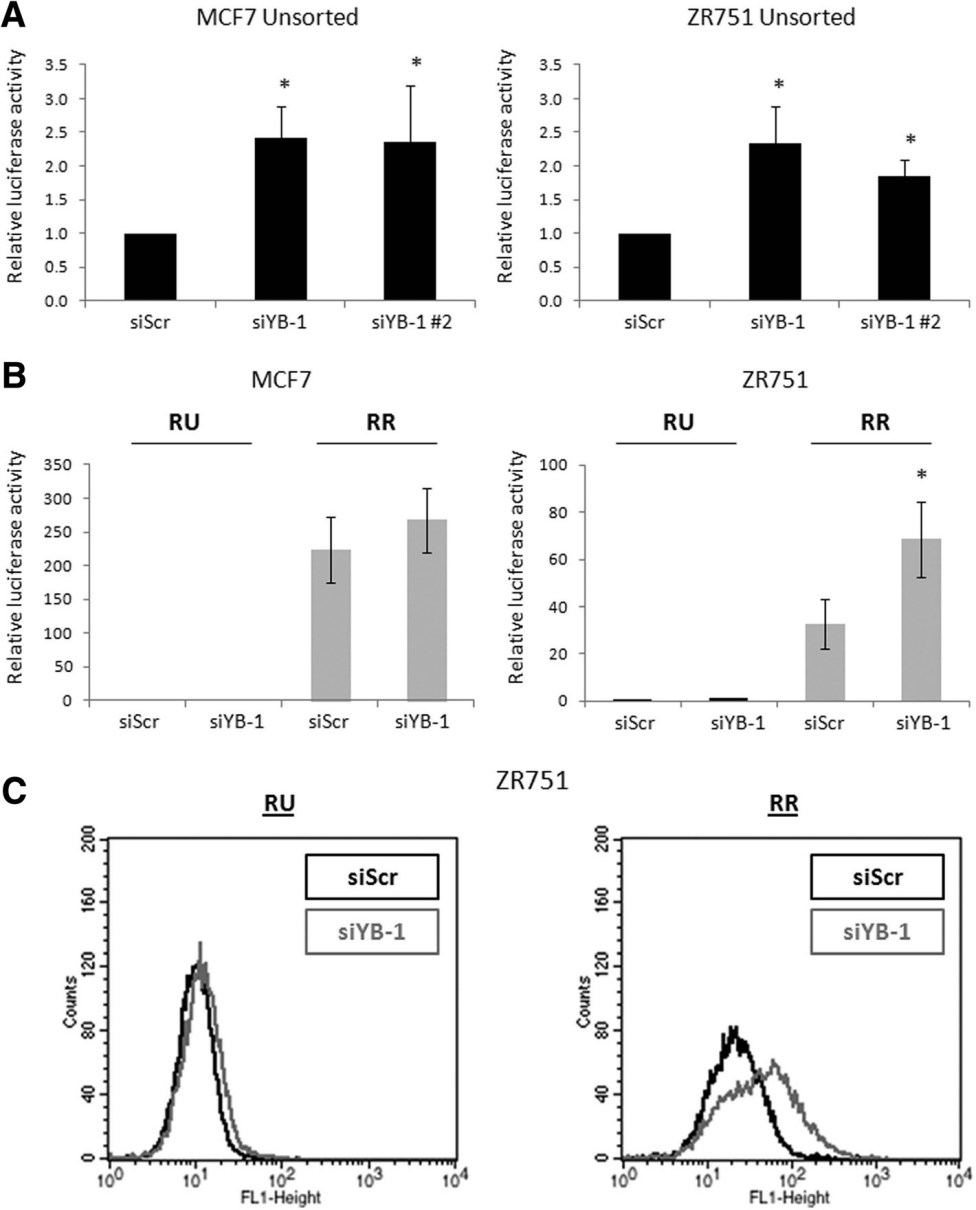
**YB-1 regulates the Sox2 transcription reporter activity only in the RR cell subset. (A)** Luciferase assay results of Sox2 reporter luciferase activity in MCF7 Unsorted cells (with natural proportions of RU and RR cells) after 72-hour treatments of scrambled or 2 unique YB-1 siRNAs at 20 nM. **(B)** Luciferase assay data showing Sox2 reporter luciferase activity relative to the RU siRNA treatment luciferase value and **(C)** flow cytometry analyses of Sox2 reporter GFP expression in MCF7 and ZR751 RU and RR cells after 72-hour 20 nM scrambled or YB-1 siRNA treatments.

Next, we asked if the Sox2 reporter activity is also regulated by YB-1. Unsorted cells derived from MCF7 and ZR751, which stably express the Sox2 reporter, showed significantly increased luciferase activity in response to YB-1 siRNA knockdown (Figure 4A). When we performed the same experiment using purified RU and RR cells, we found that YB-1 knockdown induced luciferase activity and GFP expression in the RR cells (Figure 4B and C). The small increase seen in the MCF7 RR cells (Figure 4B) could be due to the innately high luciferase activity in that cell population. In contrast, the luciferase activity and GFP expression of RU cells remained barely detectable after siRNA knockdown of YB-1 (Figure 4B and C), despite a substantial increase in Sox2 protein (Figure 1B).

### YB-1 knockdown induces differential gene expression patterns in RU and RR cells

**Figure 5:**
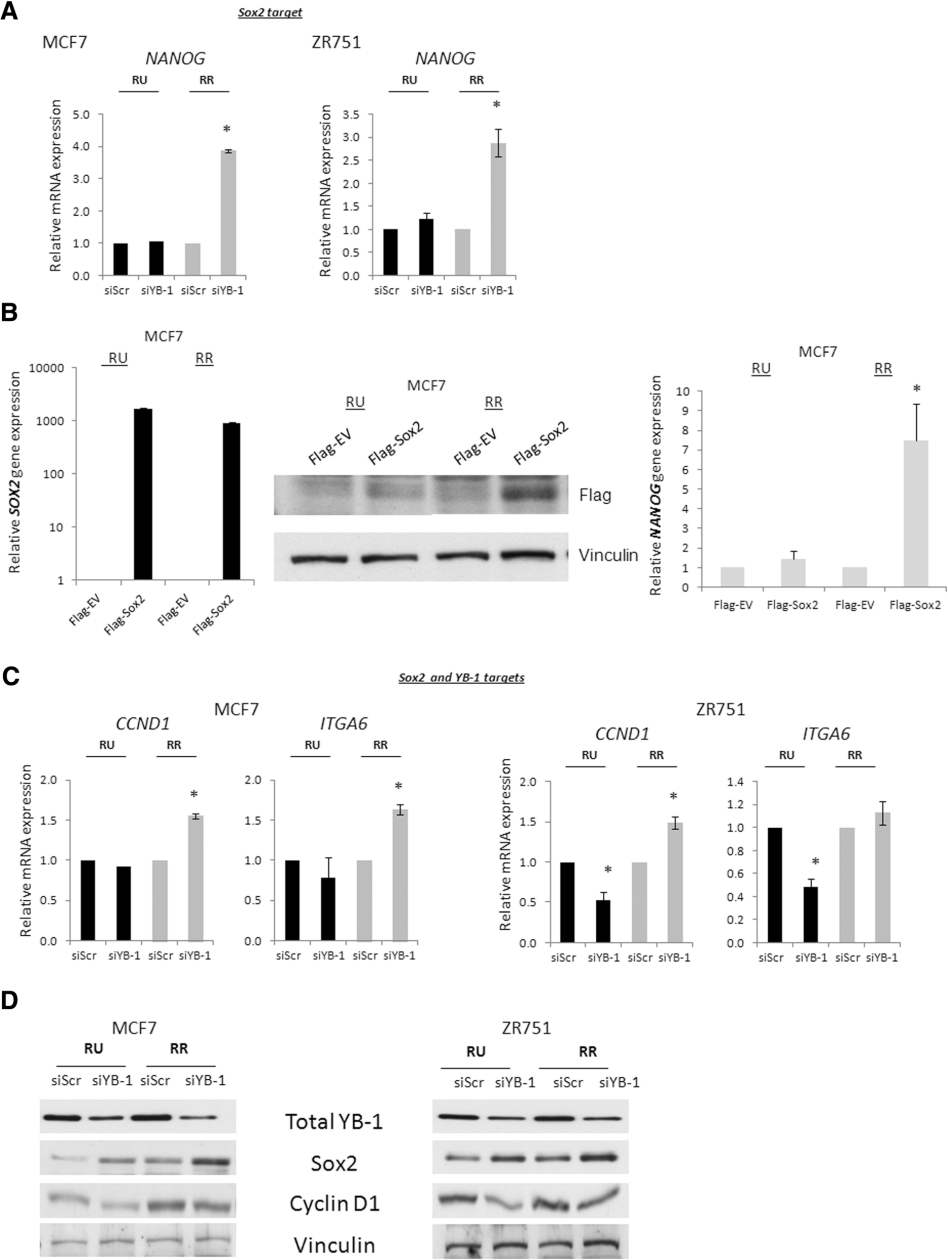
**YB-1 knockdown induces different gene expression patterns in RU and RR cells. (A)** Quantitative-RT-PCR analyses of relative mRNA transcripts of Sox2 only target *NANOG* in MCF7 and ZR751 RU and RR cells after scrambled or YB-1 siRNA treatment at 20 nM for 72 hours. **(B)** MCF7 RU and RR cells were transfected with 3 μg of pcDNA3-Flag-Empty Vector (EV) or pcDNA3-Flag-Sox2 and harvested for mRNA after 72 hours. Q-PCR analyses was performed using primers designed against *SOX2* and *NANOG. SOX2* transcript levels are normalized to the Flag-EV transfections. Accompanying Flag western blot is shown. **(C)** Quantitative-RT-PCR analyses of Sox2 and YB-1 targets *CCND1* (Cyclin D1), and *ITGA6* (CD49f) in MCF7 and ZR751 RU and RR cells after scrambled or YB-1 siRNA treatment at 20 nM for 72 hours and **(D)** accompanying western blot showing YB-1, Sox2 and Cyclin D1 protein expression.

As we have demonstrated that YB-1 can increase the Sox2 reporter activity in RR but not RU cells, we questioned if this difference may result in differential regulation of Sox2 downstream target genes between these two cell subsets, which may contribute to their phenotypic differences. To address this question, we selected a panel of genes that are known to be up-regulated by Sox2, or by both YB-1 and Sox2. For genes that have been shown to be up-regulated by Sox2 but not YB-1, such as *NANOG*[35], YB-1 knockdown is expected to result in no detectable change in *NANOG* expression in RU cells, since the Sox2 reporter activity is undetectable in these cells. In contrast, the same experimental manipulation, which resulted in a compensatory increase in Sox2 expression and activity in RR cells, is expected to up-regulate the expression of *NANOG*. In keeping with this concept, the expression level of *NANOG* did not change appreciably in response to YB-1 knockdown in RU cells derived from MCF7 and ZR751 cells; in contrast, the same treatment resulted in a significant increase in the *NANOG* expression level in RR cells derived from the two cell lines (Figure 5A). To further show that increased Sox2 protein expression induces Sox2 activity in the MCF7 cells despite a marginal increase as detected by luciferase (Figure 4B), we overexpressed Sox2 in RU and RR cells derived from MCF7, and detected an increase expression of the *NANOG* transcripts only in the RR cells (Figure 5B).

For genes that are known to be up-regulated by both YB-1 and Sox2, such as *CCND1* (Cyclin D1) [12,36] and *ITGA6* (CD49f) [25], YB-1 knockdown is expected to decrease the expression of these two genes in RU cells; in contrast, YB-1 knockdown should result in a compensatory increase in Sox2 and downstream gene expression, thus RR cells should sustain or increase the expressions of these genes. In keeping with this concept, the transcript levels of both *CCND1* and *ITGA6* were decreased or stayed the same in RU cells in response to YB-1 knockdown (Figure 5C), and the levels of *CCND1* and *ITGA6* were elevated after YB-1 knockdown in RR cells (Figure 5C, Additional file 1: Figure S4). Correlating with our mRNA data, western blotting showed that YB-1 knockdown decreased Cyclin D1 protein expression in RU cells derived from both cell lines, whereas the treatment induced no appreciable change in the protein expression of Cyclin D1 in RR cells (Figure 5D).

### Up-regulation of Sox2 and its downstream targets is accompanied by enhanced tumorigenic properties in YB-1 down-regulated RR cells

**Figure 6:**
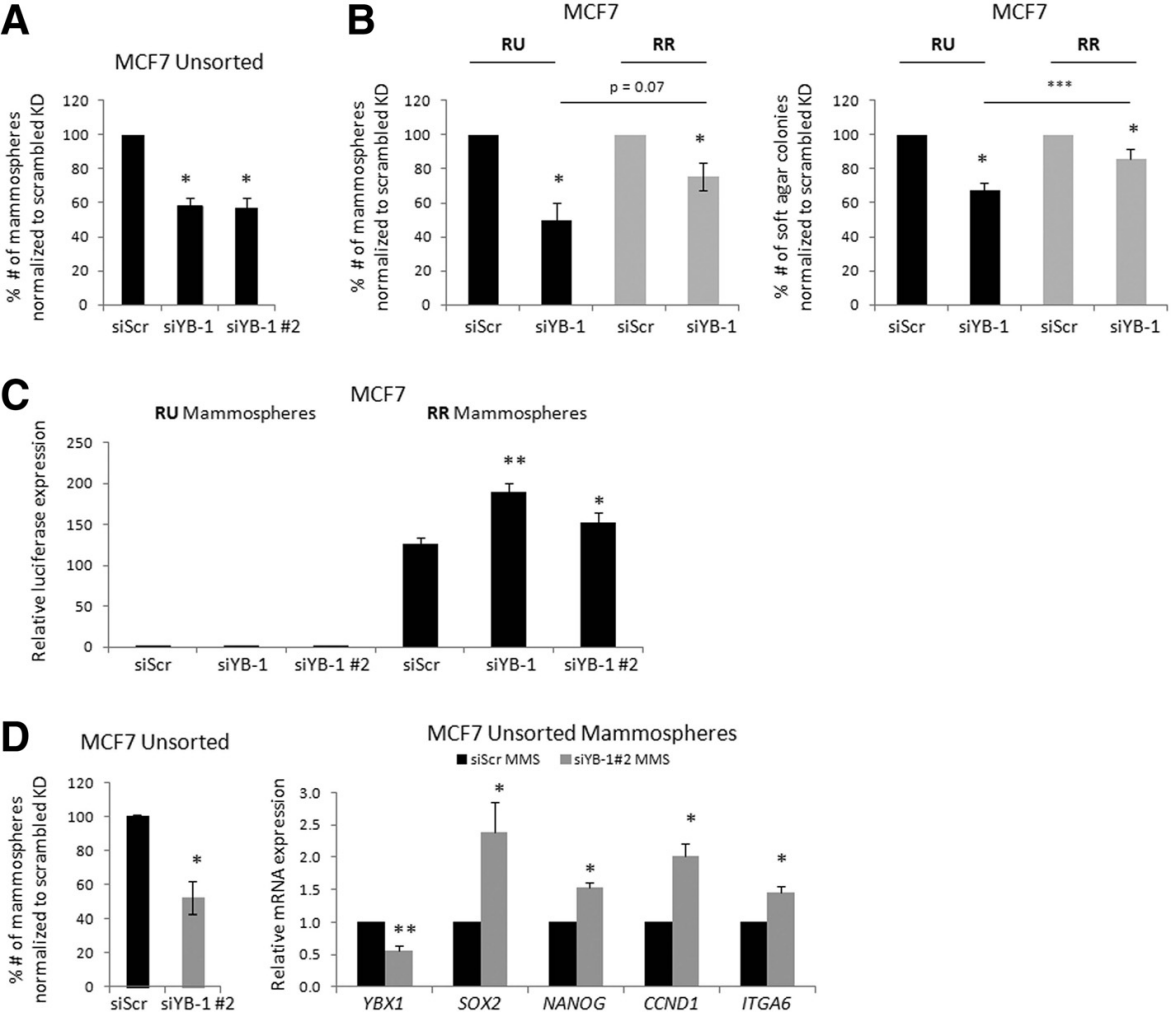
**Up-regulation of Sox2 and its downstream targets is accompanied by enhanced tumorigenic properties in YB-1 down-regulated RR cells. (A)** Mammosphere assay formation efficiency of MCF7 Unsorted cells after 72-hour 20 nM of scrambled or YB-1 siRNAs. **(B)** Mammosphere and soft agar colony forming efficiency of MCF7 RU and RR cells after 72-hour 20 nM of scrambled or YB-1 siRNAs. **(C)** Luciferase assay results of collected MCF7 RU and RR 7-day mammospheres formed after 72-hour 20 nM of scrambled or YB-1 siRNAs normalized to the RU cells siScr treatment luciferase value. **(D)** Mammosphere assay formation efficiency of MCF7 Unsorted cells after 72-hour 20 nM treatment of scrambled or YB-1 siRNA #2, and accompanying quantitative-RT-PCR analyses of relative mRNA transcripts of *YBX1* (YB-1)*, SOX2*, *NANOG, CCND1* (Cyclin D1), and *ITGA6* (CD49f) from resulting mammospheres after 7-day mammosphere culture and previous 72-hour 20 nM scrambled or YB-1 siRNA #2. YB-1 siRNA #2 was used here as it showed superior knockdown efficiency in the 7-day mammosphere culture conditions.

Next, we asked if RU and RR cells exhibit differential phenotypes in response to YB-1 down-regulation. It has been previously reported that YB-1 knockdown can significantly decrease mammosphere formation in BC cells [25]. We found similar findings for MCF7 and ZR751 after siRNA knockdown of YB-1; the reduction for MCF7 was approximately 50% (Figure 6A) and that for ZR751 was approximately 20% (Additional file 1: Figure S5). Using our sorted RU and RR cells derived from MCF7, we found that YB-1 knockdown reduced the number of mammospheres and soft agar colonies formed in RR cells significantly less efficiently (20% reduction) as compared to RU cells (40% reduction) (Figure 6B). Similar findings were observed for ZR751 (Additional file 1: Figure S5). Pictures of MCF7 mammospheres and soft agar are shown in Additional file 1: Figure S6.

To test if the lesser impact of YB-1 knockdown on the mammosphere formation of RR cells is directly related to the compensatory increase in Sox2 expression and activity in these cells, we collected the mammospheres derived from RU and RR cells treated with YB-1 siRNA. As shown in Figure 6C, we observed an increase in luciferase activity in the remaining mammospheres after YB-1 knockdown derived from RR cells but not RU cells (Figure 6C). Further, we analyzed the gene expression of the collected mammospheres derived MCF7 ‘Unsorted’ cells treated with YB-1 siRNA. As shown in Figure 6D, we found significantly increased expression levels of *SOX2*, *NANOG, CCND1* and *ITGA6* in these cells*,* a pattern that is similar to that of RR cells treated with YB-1 siRNA (Figure 5)*.* Parallel findings were observed for MCF7 parental cells (Additional file 1: Figure S7).

## Discussion

While the functional importance of Sox2 in embryonic stem cells is well characterized, the biological significance of Sox2 in cancer has not been extensively studied. Sox2 expression is well characterized in embryonic stem cells [1], where Sox2 is regulated by the Wnt, BMP, and JAK-STAT signaling pathways [1]. Aberrant expression of Sox2 in cancer cells has been well documented in a number of tumor types, but the mechanisms underlying this biochemical aberrancy is largely unexplored. In BC, there is evidence that Sox2 carries biological significance [11,12,15], although how Sox2 overexpression is regulated in this cell type is unknown. We hypothesized that YB-1, another transcription factor important in stem cell biology and the pathogenesis of BC [22,25,37], regulates Sox2 in BC cells.

Our findings led us to conclude that YB-1 regulates the expression of Sox2 in BC, likely at the transcriptional level. This conclusion is supported by our observations that knockdown of YB-1 substantially up-regulated the *SOX2* transcripts and protein expression in MCF7 and ZR751. Furthermore, using the ChIP assay, we also found evidence that YB-1 interacts with the proximal promoter of *SOX2,* which contains multiple YB-1 binding consensus sequences. We were surprised with the finding that YB-1 negatively regulates Sox2, since a previous report has described a positive correlation between YB-1 and Sox2 in glioma cells [37]. It is likely that the regulatory relationship between these two important stem cell transcription factors is complex and the discrepancy between positive or negative regulation is cell type-specific.

Our data also led us to conclude that activation of YB-1, as evidenced by the phosphorylation of YB-1 at serine-102, is required to regulate Sox2 expression. Specifically, down-regulation of YB-1 activation by serine phosphorylation at residue 102 by the treatment of pharmacologic inhibitors effectively increased Sox2 expression. The concept that activated YB-1 can suppress Sox2 expression correlates well with the previous observations, in which YB-1 phosphorylation at the serine-102 residue is a necessary condition for YB-1 to exert transcriptional control over its downstream gene targets, many of which are stem cell genes [22,25]. This concept also correlates with the previous findings that YB-1 activation, known to be mediated by kinases such as Akt, RSK1/2, and GSK3ß, confers its ability to translocate to the nucleus, bind to various gene promoters, and regulate their expression in both ER- positive and negative BC cells [31-33]. While we understand that our experiments in this study involves the use of pharmacologic inhibitors that carry some degree of non-specificity, results of these studies are in parallel with those derived from studies using YB-1 siRNAs.

Based on our findings that YB-1 suppresses the expression of Sox2 in BC, and the previous observation that ER-negative BC cell lines generally have a higher level of YB-1 activation (phosphorylation of YB-1 at serine 102) than ER-positive BC cell lines [22], we speculated that the expression level of Sox2 is higher in ER-positive BC cells. To this end, we reviewed published cDNA gene expression microarray data collected from a comprehensive panel of 50 BC cell lines [34]. Indeed, we found that ER-positive BC cell lines expressed a significantly higher *SOX2* expression than ER-negative cell lines (Additional file 1: Figure S1). Similar observations were made in our previously published study using western blotting [16]. Of note, other YB-1 positively regulated genes such as *CD44*, *MET*, and *EGFR* are likewise previously reported to be highly expressed in ER-negative BC compared to ER-positive BC [38]. Further, in support that YB-1 also suppresses Sox2 expression in ER-negative BC, we demonstrated that YB-1 knockdown increased Sox2 expression in MDA-MD-231, an ER-negative BC cell line (Additional file 1: Figure S3).

While Sox2 protein expression was negatively regulated by YB-1 in both ER-positive BC cell lines in our studies, it appears that YB-1 only regulates Sox2 transcription activity in a small subset of these cells. Specifically, we found that the siRNA knockdown of YB-1 resulted in increased luciferase and GFP expression in the RR cell subset (in monolayer and in mammosphere culture) but not the RU cell subset. Our observation that the marked increase in Sox2 protein level in RU cells after YB-1 knockdown failed to induce detectable Sox2 transcription activity is in keeping with our previous observation, in which enforced expression of Sox2 in MCF7 and ZR751 using an retroviral Sox2 expression vector also failed to induce detectable Sox2 transcription activity [16]. Thus, it appears that RU and RR cells are inherently biologically different. The mechanisms underlying this phenotypic difference between these two cell subsets are currently under active investigation in our laboratory.

The drastic difference in the relationship between YB-1 and Sox2 in the two cell subsets of BC is expected to result in substantial biochemical differences, which likely underlie the phenotypic differences between RU and RR cells described previously by our group [16]. In this regard, differential results from our gene expression analyses in the RU and RR cell populations after YB-1 knockdown support this view. Specifically, following YB-1 knockdown, stem cell genes *NANOG* and *ITGA6* (CD49f) were unchanged or down-regulated in RU cells but up-regulated in RR cells. Our results strongly suggest that, with inhibition of YB-1, RU and RR cells will undergo dramatically different biochemical changes, with the stem cell-associated genes being unchanged or suppressed in RU cells whereas the expressions of these genes in RR cells are increased or sustained due to the compensatory increase in Sox2 expression and transcription activity.

Correlating with these observations, we observed that the efficiency of mammosphere formation remained relatively high in RR cells after YB-1 siRNA knockdown. Based on our findings, it is tempting to speculate that the higher efficiency of mammosphere formation in RR cells after YB-1 knockdown is due to the increased Sox2 transcription activity and the sustained expression of various Sox2 downstream target genes in these cells. In parallel with these findings, we showed that the mammospheres derived from Unsorted and parental MCF7 cells treated with YB-1 knockdown exhibited a similar gene expression pattern of RR cells treated with YB-1 knockdown, with high expression levels of *SOX2, NANOG, CCND1* and *ITGA6*.

The existence of tumor heterogeneity, as highlighted by RU and RR cell subsets in our models, may provide explanations to tumor resistance to cancer treatments. Based on the concept generated from this current study, treatments that result in YB-1 inhibition in cancer cells may up-regulate Sox2 expression and transcription activity in the RR cell subset. As a result, stem cell-related genes and possibly the stem cell phenotype can be increased or sustained in this small cell subset, leading to their persistent survival during the course of cancer treatment. Directly relevant to our discussion, at the time of writing, we are aware of an on-going NIH/NCI clinical trial examining the efficacy of Akt inhibitor MK2206 in BC patients. MK2206, like LY294002 inhibits Akt phosphorylation/activation, and we hypothesize that the inhibition of phosphorylation of YB-1 at Ser-102 will up-regulate Sox2 expression.

## Conclusions

In summary, we have characterized a novel regulatory relationship between YB-1 and Sox2, two important cancer and/or stem cell transcription factors that have been implicated in the pathogenesis of BC. In addition, in the context of the inherent dichotomy of BC cells (i.e. RU and RR cells), YB-1 contributes to the phenotypic heterogeneity between the cell subsets by mediating differential gene regulation of Sox2 downstream targets. This level of tumor heterogeneity may underline some of the mechanisms of drug resistance in BC.

## Competing interests

The authors declare no conflicts of interest.

## Authors’ contributions

KJ conceived and designed the research plan, performed experiments, analyzed data, and wrote the manuscript. FW, PW, and XY established the MCF7 and ZR751 Unsorted, RU, and RR lines. BSA provided intellectual input and critical reading of the manuscript. RL conceived and designed the research plan and wrote the manuscript. All authors read and approved the final manuscript.

## Pre-publication history

The pre-publication history for this paper can be accessed here:

http://www.biomedcentral.com/1471-2407/14/328/prepub

## Supplementary Material

Additional file 1: Figure S1 Sox2 is expressed higher in ER-positive breast cancer cell lines. Published gene expression microarray data from 50 BC cell lines reveals relative average Sox2 expression levels with respect to estrogen receptor status. **Figure S2.** Sox2 does not modulate YB-1 expression or phosphorylation at serine-102. Western blot of Sox2, phospho-YB-1^Ser102^, and total YB-1 protein expression in MCF7 and ZR751 RU and RR cells after 72-hour 20nM scrambled or Sox2 siRNA treatments. **Figure S3.** YB-1 knockdown increases Sox2 transcript levels in ER-MDA-MB-231 cells. Quantitative-RT-PCR analyses of *SOX2* mRNA in MDA-MB-231 RU and RR cells after 72-hour 20 nM scrambled or YB-1 siRNA treatments. **Figure S4.** YB-1 siRNA #2 treatments result in up-regulation of *SOX2* and *CCND1*. Quantitative-RT-PCR analyses of relative *CCND1*, *YBX1*, and *SOX2* mRNA in MCF7 RU and RR cells after 72-hour 20 nM scrambled or YB-1 siRNA #2 treatments. **Figure S5.** ZR751 RR cells form more mammospheres after YB-1 knockdown compared to ZR751 RU cells. Mammosphere assay formation efficiency of ZR751 Unsorted, RU, and RR cells after 72-hour 20 nM scrambled or YB-1 siRNA treatments. **Figure S6.** Mammospheres and soft agar colonies photographs after YB-1 knockdown in MCF7 RU and RR cells. Mammosphere assay formation (Day 7) and soft agar colony formation (Day 14) of MCF7 RU and RR cells after 72 hour treatments of 20 nM scrambled or YB-1 siRNAs. **Figure S7.** Mammospheres derived from YB-1 down-regulated MCF7 Parental cells show up-regulation of *SOX2* and other targets. Mammosphere assay formation efficiency of MCF7 Parental cells after 72-hour 20 nM scrambled or YB-1 siRNA #2 treatments, and quantitative-RT-PCR analyses of *YBX1* (YB-1)*, SOX2*, *NANOG, CCND1*, and *ITGA6* mRNA from resulting mammospheres after 7-day mammosphere culture and 72-hour 20 nM scrambled or YB-1 siRNA #2. YB-1 siRNA #2 was used here for superior knockdown efficiency in the 10-day assay. Click here for file
