# Supplementary material for: YB-1 regulates Sox2 to coordinately sustain stemness and tumorigenic properties in a phenotypically distinct subset of breast cancer cells
Source: BMC Cancer. 2014 May 9;14:328. doi: 10.1186/1471-2407-14-328 (PMC4025193; doi:10.1186/1471-2407-14-328)

**Jung et al. YB-1 regulates Sox2 to coordinately sustain stemness and tumorigenic properties in a phenotypically distinct subset of breast cancer cells.**

**Supplementary Figures:**
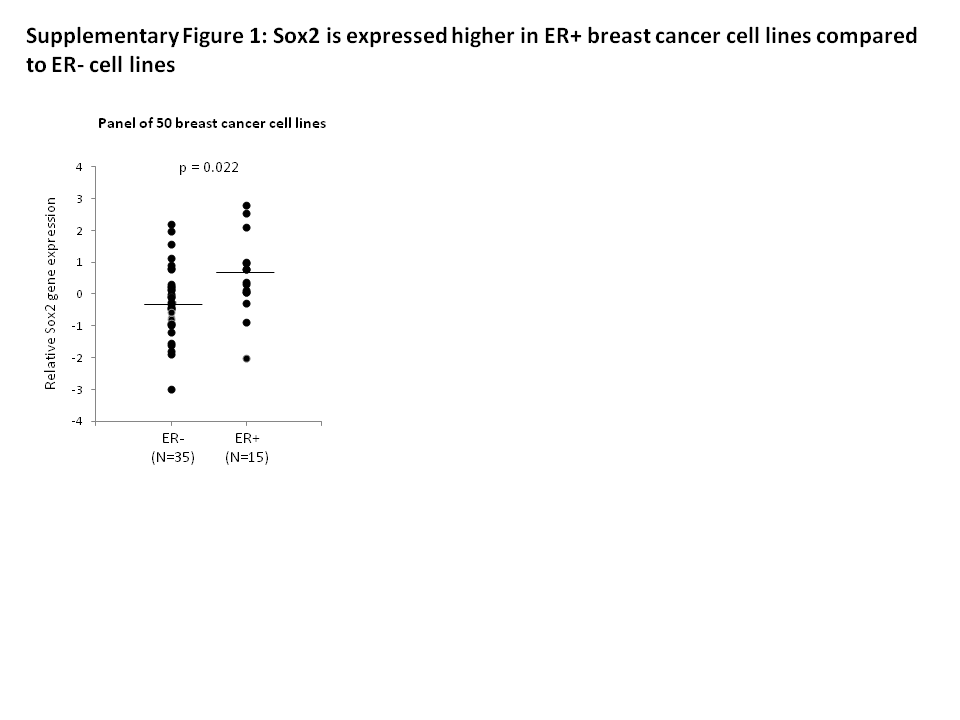

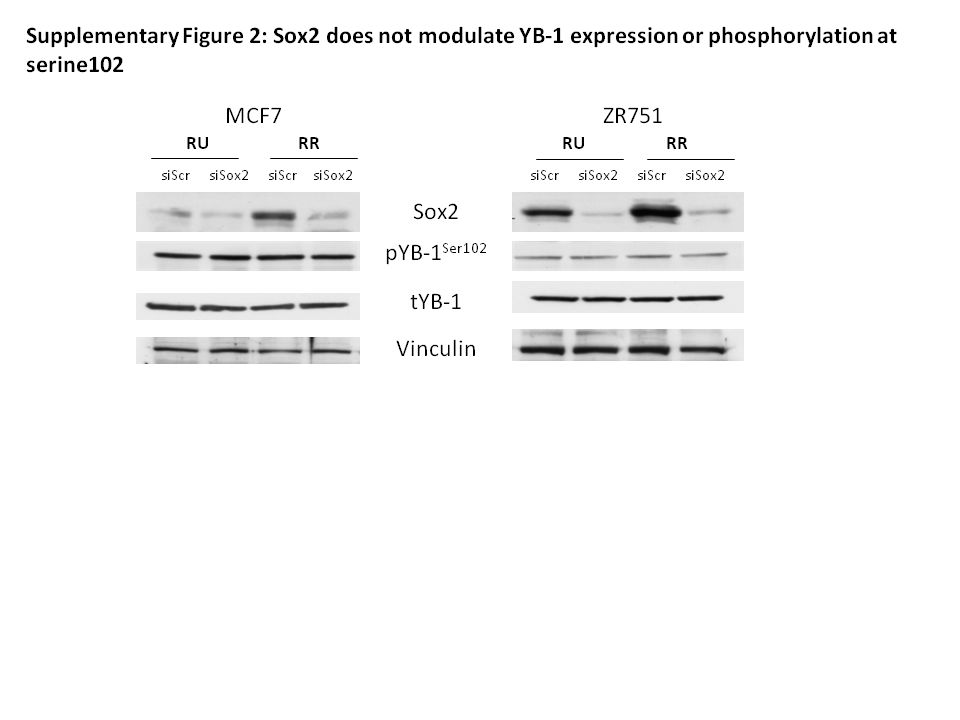

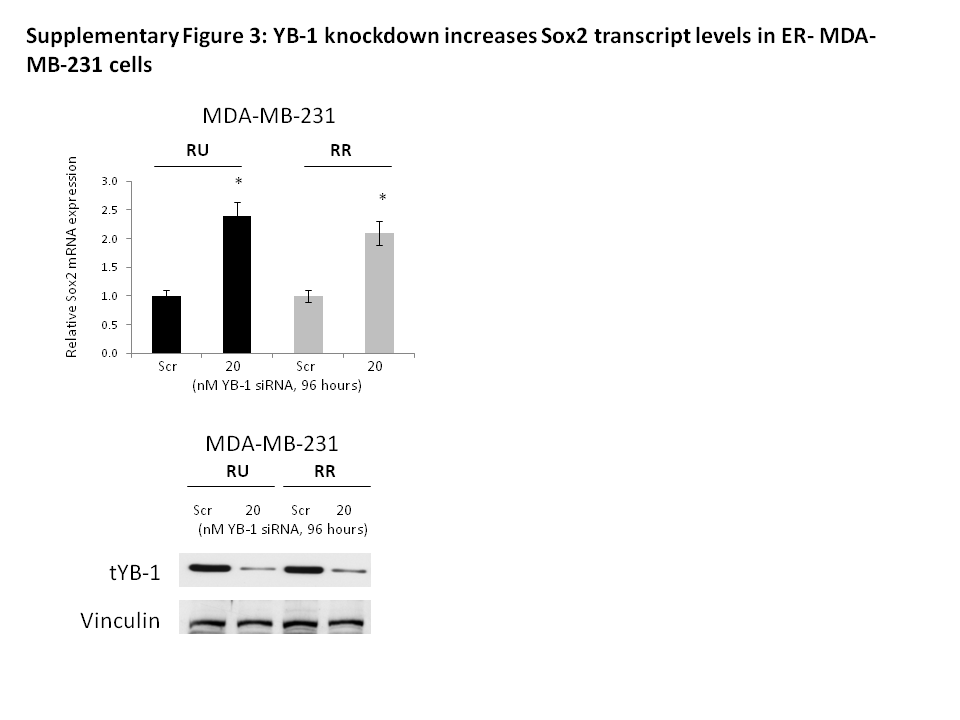

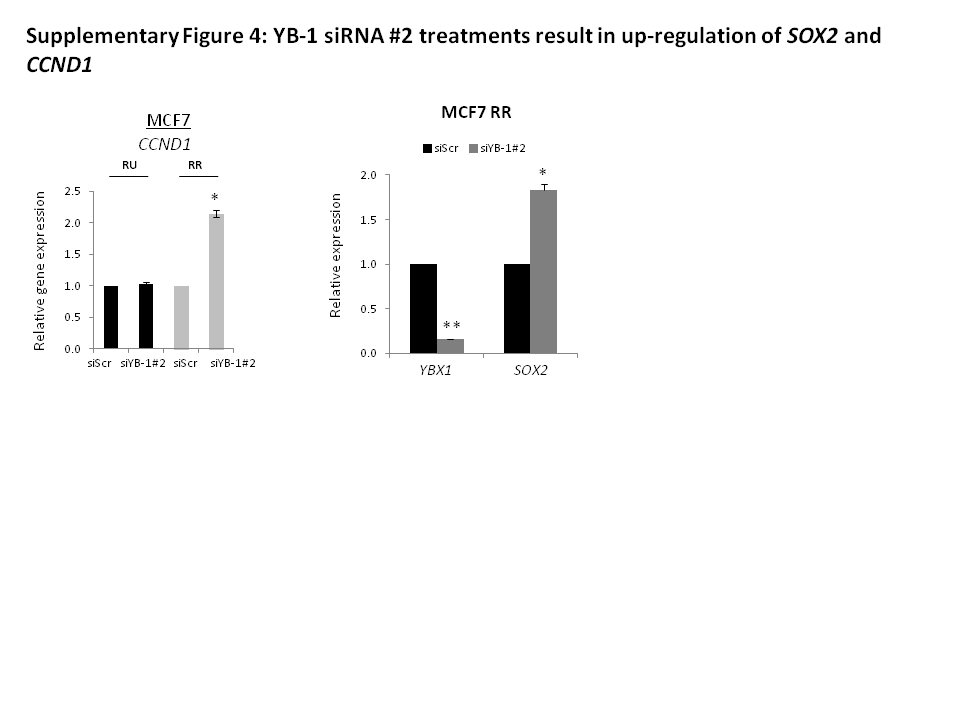

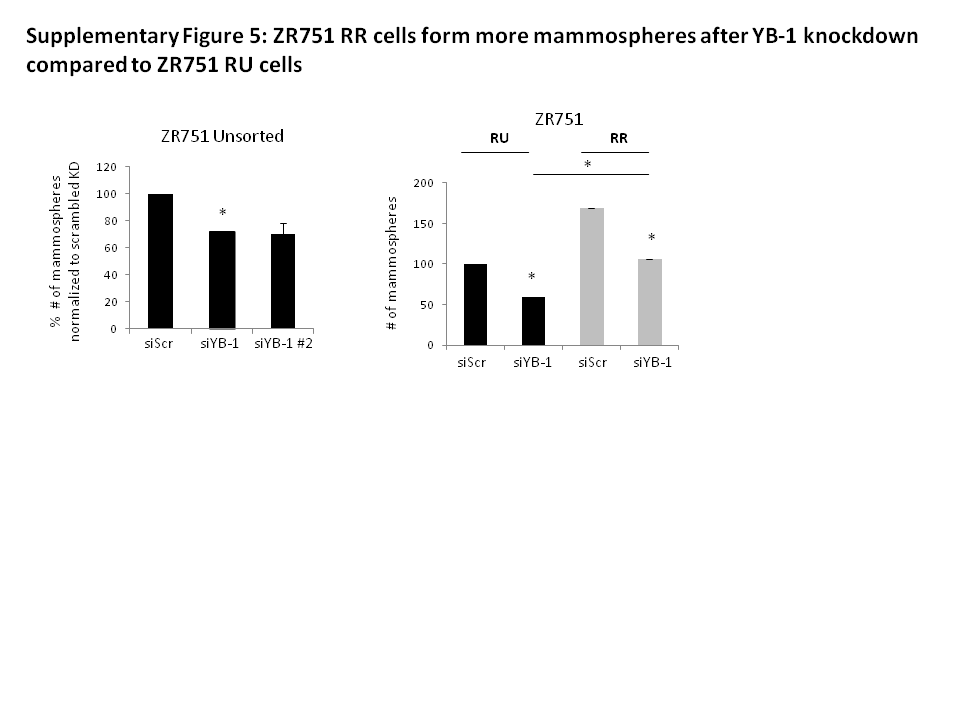

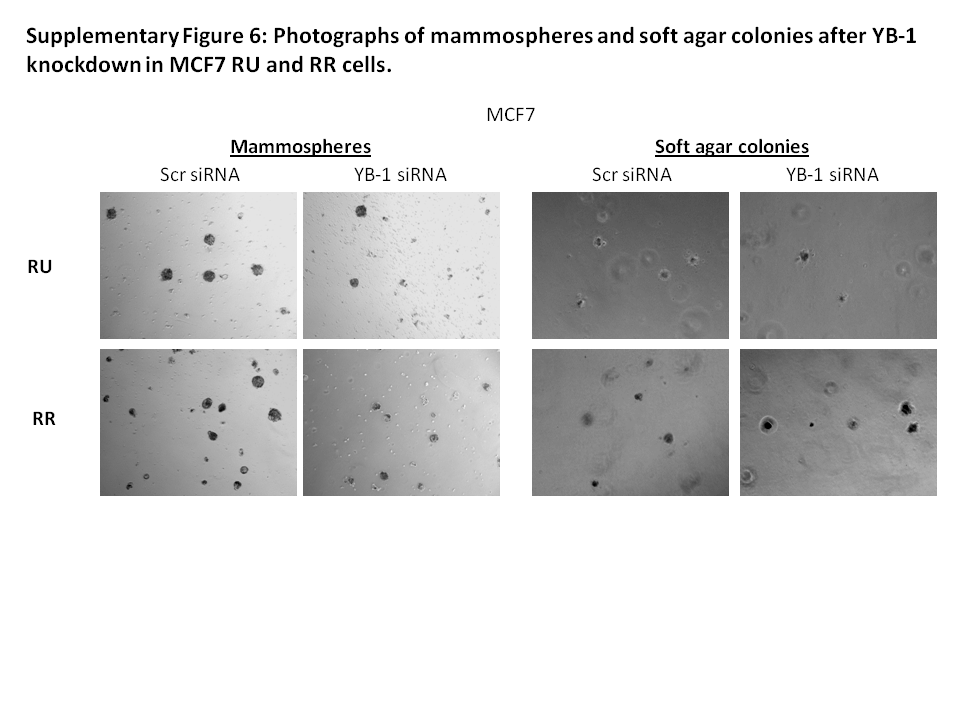

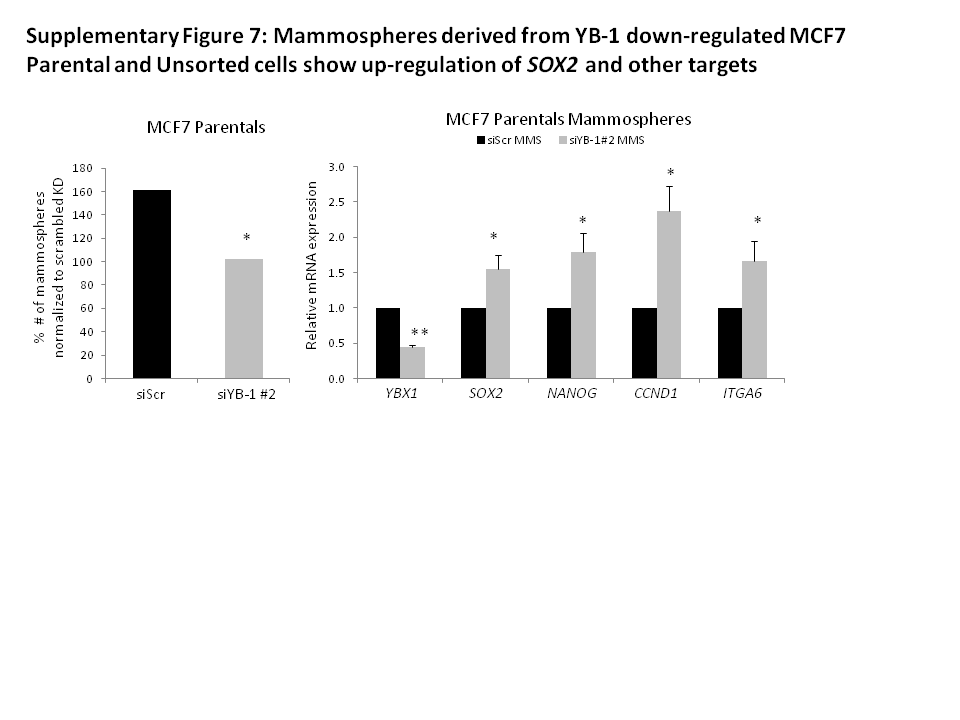

Supplement: Additional file 1: Figure S1 — Sox2 is expressed higher in ER-positive breast cancer cell lines. Published gene expression microarray data from 50 BC cell lines reveals relative average Sox2 expression levels with respect to estrogen receptor status. Figure S2. Sox2 does not modulate YB-1 expression or phosphorylation at serine-102. Western blot of Sox2, phospho-YB-1Ser102, and total YB-1 protein expression in MCF7 and ZR751 RU and RR cells after 72-hour 20nM scrambled or Sox2 siRNA treatments. Figure S3. YB-1 knockdown increases Sox2 transcript levels in ER-MDA-MB-231 cells. Quantitative-RT-PCR analyses of SOX2 mRNA in MDA-MB-231 RU and RR cells after 72-hour 20 nM scrambled or YB-1 siRNA treatments. Figure S4. YB-1 siRNA #2 treatments result in up-regulation of SOX2 and CCND1. Quantitative-RT-PCR analyses of relative CCND1, YBX1, and SOX2 mRNA in MCF7 RU and RR cells after 72-hour 20 nM scrambled or YB-1 siRNA #2 treatments. Figure S5. ZR751 RR cells form more mammospheres after YB-1 knockdown compared to ZR751 RU cells. Mammosphere assay formation efficiency of ZR751 Unsorted, RU, and RR cells after 72-hour 20 nM scrambled or YB-1 siRNA treatments. Figure S6. Mammospheres and soft agar colonies photographs after YB-1 knockdown in MCF7 RU and RR cells. Mammosphere assay formation (Day 7) and soft agar colony formation (Day 14) of MCF7 RU and RR cells after 72 hour treatments of 20 nM scrambled or YB-1 siRNAs. Figure S7. Mammospheres derived from YB-1 down-regulated MCF7 Parental cells show up-regulation of SOX2 and other targets. Mammosphere assay formation efficiency of MCF7 Parental cells after 72-hour 20 nM scrambled or YB-1 siRNA #2 treatments, and quantitative-RT-PCR analyses of YBX1 (YB-1), SOX2, NANOG, CCND1, and ITGA6 mRNA from resulting mammospheres after 7-day mammosphere culture and 72-hour 20 nM scrambled or YB-1 siRNA #2. YB-1 siRNA #2 was used here for superior knockdown efficiency in the 10-day assay. [file 1471-2407-14-328-S1.doc]
